# Capturing Ultraviolet Radiation Exposure and Physical Activity: Feasibility Study and Comparison Between Self-Reports, Mobile Apps, Dosimeters, and Accelerometers

**DOI:** 10.2196/resprot.9695

**Published:** 2018-04-17

**Authors:** Elke Hacker, Caitlin Horsham, Martin Allen, Andrea Nathan, John Lowe, Monika Janda

**Affiliations:** ^1^ Institute of Health and Biomedical Innovation School of Public Health and Social Work Queensland University of Technology Brisbane Australia; ^2^ Department of Electrical and Computer Engineering University of Canterbury Christchurch New Zealand; ^3^ The MacDiarmid Institute for Advanced Materials and Nanotechnology Wellington New Zealand; ^4^ Institute for Health and Ageing Australian Catholic University Melbourne Australia; ^5^ Faculty of Science, Health, Education and Engineering School of Health and Sport Sciences University of the Sunshine Coast Maroochydore Australia

**Keywords:** sun-protection, sunburn, health behaviour, health promotion, formative research

## Abstract

**Background:**

Skin cancer is the most prevalent cancer in Australia. Skin cancer prevention programs aim to reduce sun exposure and increase sun protection behaviors. Effectiveness is usually assessed through self-report.

**Objective:**

It was the aim of this study to test the acceptance and validity of a newly developed ultraviolet radiation (UVR) exposure app, designed to reduce the data collection burden to research participants. Physical activity data was collected because a strong focus on sun avoidance may result in unhealthy reductions in physical activity. This paper provides lessons learned from collecting data from participants using paper diaries, a mobile app, dosimeters, and accelerometers for measuring end-points of UVR exposure and physical activity.

**Methods:**

Two participant groups were recruited through social and traditional media campaigns 1) Group A—UVR Diaries and 2) Group B—Physical Activity. In Group A, nineteen participants wore an UVR dosimeter wristwatch (University of Canterbury, New Zealand) when outside for 7 days. They also recorded their sun exposure and physical activity levels using both 1) the UVR diary app and 2) a paper UVR diary. In Group B, 55 participants wore an accelerometer (Actigraph, Pensacola, FL, USA) for 14 days and completed the UVR diary app. Data from the UVR diary app were compared with UVR dosimeter wristwatch, accelerometer, and paper UVR diary data. Cohen kappa coefficient score was used to determine if there was agreement between categorical variables for different UVR data collection methods and Spearman rank correlation coefficient was used to determine agreement between continuous accelerometer data and app-collected self-report physical activity.

**Results:**

The mean age of participants in Groups A (n=19) and B (n=55) was 29.3 and 25.4 years, and 63% (12/19) and 75% (41/55) were females, respectively. Self-reported sun exposure data in the UVR app correlated highly with UVR dosimetry (κ=0.83, 95% CI 0.64-1.00, *P*<.001). Correlation between self-reported UVR app and accelerometer-collected moderate to vigorous physical activity data was low (ρ=0.23, *P*=.10), while agreement for low-intensity physical activity was significantly different (ρ=-0.49, *P*<.001). Seventy-nine percent of participants preferred the app over the paper diary for daily self-report of UVR exposure and physical activity.

**Conclusions:**

This feasibility study highlights self-report using an UVR app can reliably collect personal UVR exposure, but further improvements are required before the app can also be used to collect physical activity data.

## Introduction

In the United States, the number of new cases of melanoma is predicted to rise from 70,000 in 2007-2011 to 116,000 in 2026-2031 [1], and similar increases are expected in other countries around the world. Ultraviolet radiation (UVR) is the main environmental risk factor for melanoma. Accurate measurement of UVR exposure is important for skin cancer prevention studies, which aim to reduce peoples’ sun exposure. Monitoring physical activity levels is also important in skin cancer prevention studies as three large-scale cross-sectional studies have shown increased levels of physical activity among adults were associated with higher levels of sunburn [2-5].

**Figure 1:**
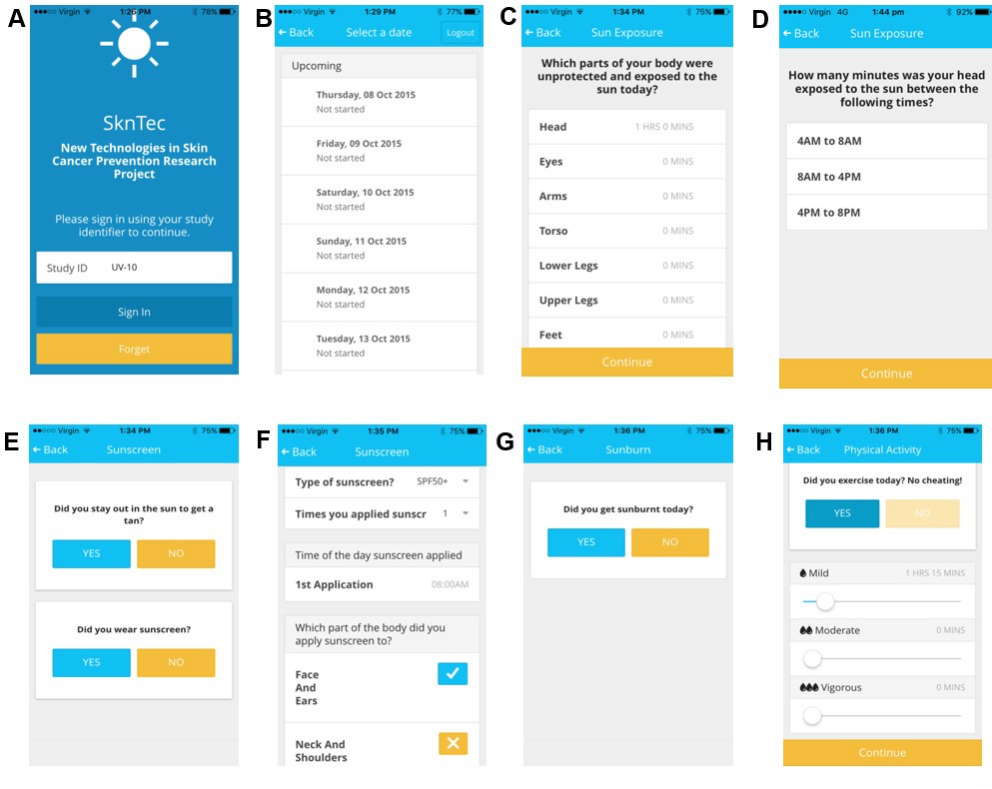
Ultralight radiation diary app. A) Log-in screen for participants to enter their unique study identifier. B) Home screen. In the home screen, participants select the date to enter their sun exposure and sun protection used for that day. The app will not let participants enter their data for the days ahead. They can only enter data for the current day or previous days. C) The participant enters which parts of the body were unprotected and exposed to the sun. In this image, the participant has specified that the head was exposed for 1 hour. D) Once a body site is selected, the next screen asks participants how many minutes they were exposed to the sun for each timeblock: 4am- 8am, 8am-4pm, and 4pm-8pm. E) The participant selects “yes” or “no”, depending on whether they stayed in the sun to get a tan and whether they wore sunscreen for the day. If a participant selects “yes” to the sunscreen question, the panel F screen appears, which details the sunscreen sun protection factor (SPF), number of times applied, time of day applied, and area of application to the body, for each application. F) This screen illustrates a participant that applied SPF 50+ sunscreen once at 8am to their face and ears. Users can scroll down to select different parts of the body where sunscreen was applied. G) The participant selects “yes” or “no”, depending on whether they were sunburnt that day. H) The participant selects “yes” or “no”, depending on whether they excercised that day, recording the duration and level of activity as mild, moderate, or vigorous.

UVR exposure data can be collected via direct observation, UVR dosimeters, or self-report, particularly for current or recent exposures. Objective measures of chronic/cumulative UVR exposure are silicone casts of the dorsum of the hand [6], DNA mutation loads of eye lids [7], and measurements of eye conjunctival ultraviolet autofluorescence [8]. The selection of the measurement tool depends on the research question, feasibility, costs, and burden to study participants [9]. Self-reported paper UVR diaries are a common form of data collection [10]. However, there are limitations to paper diaries. For example, they can be burdensome to complete, participants may miss questions, and they do not allow for real-time monitoring of compliance. Electronic data collection could overcome some of these barriers, streaming data directly into an electronic database, thus permitting real-time monitoring of participants’ entries and generating automated reminders to input data regularly, thereby reducing missing data.

UVR dosimeter technology varies greatly ranging from low-tech solutions such as polysulphone film dosimeters [11] to electronic time-stamped dosimeters [12]. Their use is not always feasible in large-scale population studies due to cost and logistics [13]. The limitations of UVR dosimeters include the device’s requirement to be worn with a clear orientation to the sun for accurate measurements and its inability to record other context-relevant information, such as use of sunscreen or protective clothing by participants. Previous studies have shown acceptable correlation between UVR dosimeter dose and paper questionnaire–reported time outdoors [10].

Similar measurement issues apply to physical activity, which can be collected using self-reported questionnaires or via objective assessment, with the use of accelerometers as the most popular choice. It is accepted that self-report and objective measures capture distinct and complementary aspects of physical activity [14,15]. There are no clear trends in the over- or under-reporting of physical activity when comparing self-report and objective methods [16]. A systematic review of 148 studies found low correlation between self-reported and objective measurements of physical activity [17].

It was the primary aim of this study to compare UVR exposure data collected using paper diaries to those collected via a mobile app (Figure 1; Multimedia Appendix 1: UVR diary app), and compare both to objectively collected data from UVR dosimeters. A secondary aim was to compare physical activity collected via the app to data from accelerometers.

## Methods

### Recruitment

**Figure 2:**
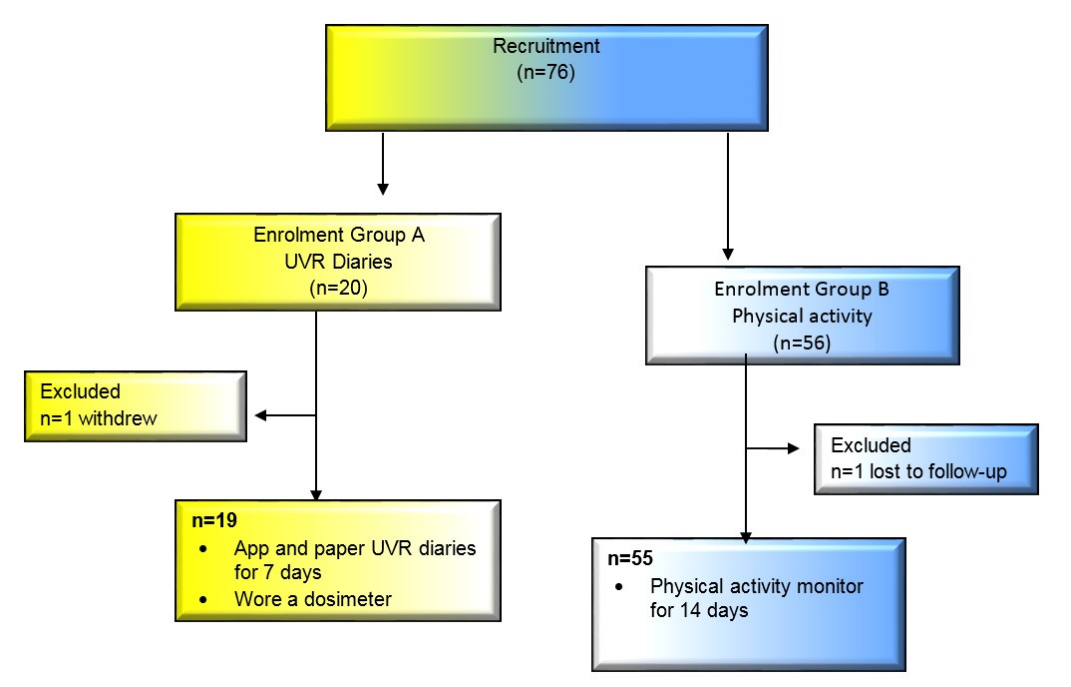
Flow chart of study participants.

Participants were recruited in Brisbane, Australia (September 2015-February 2016, during spring and summer in Australia). The Queensland University of Technology’s Human Ethics Committee approved the study and all participants gave written informed consent in line with the Declaration of Helsinki (Approval-1400000302). A convenience sample of participants were recruited using television, media, university email, social media, and flyers distributed at local sporting centers or clubs. Eligibility criteria included males and females, 18 to 35 years, who have never been diagnosed with a melanoma and own a smartphone. Participants completed an online demographic questionnaire and were recruited consecutively into 2 participant groups 1) Group A—UVR Diaries; and 2) Group B—Physical Activity (Figure 2).

### Ultraviolet Radiation Exposure Behavior

In Group A, participants recorded their sun exposure, sunburn, and physical activity levels using both the UVR app and a paper UVR diary for 7 consecutive days. During these 7 days, participants were also asked to wear an UVR dosimeter wristwatch (University of Canterbury, New Zealand) when outside. At the end of the 7-day assessment period, participants completed an audio-recorded telephone or in-person interview. This was conducted to assess in-depth the usability and convenience of the app and paper diaries. Example interview questions include: “What barriers did you experience using each of the diaries?” and “How helpful were each of the diaries to track your sun behavior?” In the interview, participants were also asked to select whether they preferred to complete a UVR diary method either 1) on paper or 2) via an app.

### Devices Used to Capture UVR Exposure

#### Ultraviolet Radiation Dosimeter

The features of the dosimeter were previously described in detail [12]. Each UVR dosimeter device was calibrated to the UVR levels in Queensland using the Australian Radiation Protection and Nuclear Safety Agency weather station data. Briefly, each device captured data for 3 hours between 11am to 2pm on a cloud-free day in an open field 100 metres from the weather station. Any device that recorded data greater than 5% outside the weather station output was adjusted and retested following the above protocol. Three dosimeters malfunctioned (3/19) during the study with data unusable when downloaded from the device by the research team. Participants were not reissued another dosimeter to replace the nonfunctioning one as the data collection period had ended.

#### Paper Ultraviolet Radiation Diaries

The paper diary (Multimedia Appendix 2) was adapted from previous studies [9,18].

#### Ultraviolet Radiation App

The app questions were modified from the paper diaries for the smaller mobile screen. Figure 1 displays each question the user is asked to complete in the app. An advantage of the app was data fields need to be completed before the user can continue to the next section.

### Physical Activity

In Group B, participants used the same UVR app as Group A and wore an Actigraph wGT3X-BT accelerometer (Actigraph, Pensacola, FL, USA) on the hip for 14 consecutive days.

#### Accelerometer

Data were processed and scored using ActiLife software (version 6.11.9) [19]. Raw data were converted into 1 minute epochs. Sufficient wear time was defined as ≥10 hours/day. Days with insufficient wear time were excluded.

### Statistical Analysis

SPSS software (version 23.0) was used to calculate Cohen kappa coefficient score to determine if there was agreement between categorical variables for different UVR data collection methods. Spearman rank correlation coefficient was used to determine correlation between accelerometer data and app-collected self-report physical activity. Values of >0.4 to 0.6 were considered moderate, >0.6 to 0.8 substantial, and >0.8 to 1.0 almost perfect agreement [20]. The qualitative data was coded into 3 themes: enablers, barriers to use, and behavior change.

Personal UVR exposure variables were dichotomised to categorical data: UVR diary app data was coded “yes” if the participant responded affirmative when asked “Did the participant report sun exposure between 8am to 4pm?” UVR dosimeter data was coded “yes” if the dose of UVR detected between 8am to 4pm was above 0.05 standard erythemal dose (SED), and the UVR dosimeter data was coded “no” if the dose of UVR detected between 8am to 4pm was below 0.01 SED. Paper diary data was coded “yes” if the participants reported any sun exposure between 8am to 4pm.

Personal physical activity variables were coded into intensity levels: UVR diary app data was coded “yes” if the participant responded affirmative when asked “Did you exercise today?” and was further coded into intensity levels based on the selection of “mild”, “moderate”, or “vigorous.” The length of time that exercise was conducted was also collected; accelerometer data between 100 to 2019 counts per minute were scored as low intensity and ≥2020 counts per minute were scored as moderate to vigorous intensity. This was done using ActiLife software (version 6.11.9) [19].

## Results

### Participant Characteristics

The mean age of participants in Groups A and B was 29.3 and 25.4 years, respectively. In Group A and B, most participants were female (12/19, 63% and 41/55, 75% respectively) and the majority had fair skin (10/19, 53% and 33/55, 60% respectively). Participant characteristics are reported in Table 1. Complete data is available for 19 participants in Group A and 55 participants in Group B. One participant in Group A (1/20, 5%) withdrew due to time constraints and 1 participant in Group B (1/56, 2%) was lost to follow-up as contact could not be re-established. All 19 participants in Group A completed 7 days of app and paper diaries, and a total of 112 days with dosimeter data were available from 16 of these participants. Forty-two per cent of participants (8/19) had 1 or more answer fields missing in the paper diary. There was no missing data in the UVR app diary. In Group B, 53 participants had sufficient accelerometer wear time and corresponding app data for at least 1 day, with on average, 7 days of objective and self-reported physical activity data available per participant (SD 3.5; total days=372).

**Table 1:** Participant characteristics. UVR: ultraviolet radiation.

Characteristics		Group A UVR diaries (n=19), n (%)	Group B physical activity (n=55), n (%)
Age mean (range 18-35)		29.3	25.4
**Gender**			
	Female	12 (63)	41 (75)
	Male	7 (37)	14 (25)
**Highest completed education**			
	Completed high school	3 (16)	10 (18)
	Trade or technical certification or diploma	2 (10)	6 (11)
	University or college degree	14 (74)	39 (71)
**Current work situation**			
	Employed full-time	9 (48)	12 (22)
	Employed part-time or casual	5 (26)	12 (22)
	Student	5 (26)	31 (56)
**Is your main job now**			
	Mainly indoors	16 (84)	49 (89)
	Mainly outdoors	0 (0)	0 (0)
	About equal amounts indoors and outdoors	3 (16)	6 (11)
**Eye color**			
	Blue or gray	8 (42)	15 (27)
	Green	3 (16)	6 (11)
	Brown	8 (42)	27 (49)
	Other	0 (0)	7 (13)
**Skin color**			
	Fair	10 (53)	33 (60)
	Medium	8 (42)	15 (27)
	Olive/Dark	1 (5)	6 (11)
	Black	0 (0)	1 (2)
**Would your skin burn in strong summer sun for 30 minutes without protection?**			
	My skin would not burn at all	3 (16)	7 (13)
	My skin would burn lightly	3 (16)	17 (31)
	My skin would burn moderately	10 (52)	18 (33)
	My skin would burn severely	3 (16)	13 (23)
**Would your skin tan if you spend several weeks at the beach and you are often in the strong sun without any protection?**			
	My skin would not tan	1 (5)	6 (11)
	My skin would tan lightly	5 (26)	10 (18)
	My skin would tan moderately	9 (48)	24 (44)
	My skin would tan deeply	4 (21)	15 (27)

**Table 2:** Overall agreement between measurements. UVR: ultraviolet radiation.

Measurement		Sun exposure, n		Cohen kappa coefficient score (95% CI)
		Yes	No	
**Did the participant report sun exposure between 8am to 4pm (yes/no, n=16)**				0.83 (0.64-1.00)
	UVR diary app	98	14	
	UVR dosimeter^a,b^	95	15	
**Did the participant report sun exposure between 8am to 4pm (yes/no, n=19)**				0.64 (0.44-0.84)
	UVR diary app	114	19	
	Paper sun diary	117	16	
**Did the participant report sunscreen use (yes/no, n=19)**				0.97 (0.93-1.00)
	UVR diary app	55	78	
	Paper sun diary	57	76	

^a^“Yes” defined by a dose of UVR detected above 0.05 standard erythemal dose, between 8am to 4pm.

^b^Missing data due to dosimeter not being worn (n=2 days; 2 participants forgot to wear their dosimeter on 1 day of their intervention).

### Ultraviolet Radiation Exposure Behavior

Self-reported unprotected UVR exposure had high agreement with dosimeter data (κ=0.83, 95% CI, 0.64-1.00,  *P*<.001, Table 2). There was moderate agreement between UVR exposure reported using the paper diary and the app (κ=0.64, 95% CI 0.44-0.84, *P*<.001). There was almost perfect agreement for sunscreen use between the app and paper formats, (κ=0.97, 95% CI, 0.93-1.00,  *P*<.001).

### Physical Activity

The Spearman rank coefficient for low-intensity physical activity collected via self-report and accelerometer was ρ=–0.488, *P*<.001, which represents low agreement. It was ρ=0.230, *P*=.10 for moderate-to-vigorous­–intensity physical activity, which represents low agreement. The mean difference in estimated minutes per day between measures was –201 minutes/day for low-intensity and –18 minutes/day for moderate-to-vigorous–intensity physical activity.

### Interviews with Participants

In the interviews, participants reported that the UVR app was easier (16/19, 84%) and quicker (on average, 6 minutes for paper and 4 minutes for app) to use. Most people preferred the app over the paper diary (15/19, 79%), and all would prefer to use the app for monitoring periods of more than 7 days. Eight out of nineteen participants (42%) experienced barriers using the app including: insufficient phone battery (1/19, 5%); app crashing (2/19, 11%); lack of internet access (1/19, 5%); or smartphone update required (1/19, 5%). Eight out of nineteen participants (42%) reported barriers for the paper diary, including no pen (4/19, 21%); no surface to write on (1/19, 5%); flipping pages to view clothing coding (4/19, 21%); and inconvenience for travel (3/19, 16%). No participants reported losing their paper diary or mobile phone. Fifty-three per cent of participants (10/19) reported that recording their UVR exposure on a daily basis made them aware and encouraged them to use more sun protection.

## Discussion

### Principal Findings

Objectively measured UVR exposure via a dosimeter and self-reported UVR exposure via an app demonstrated substantial agreement. This finding adds to the evidence base that self-report using an app can be a valid form of UVR exposure data collection. Our study results were similar to previous studies which also supported the validity of self-reported diary-collected UVR exposure compared to dosimeters [10,21-23]. An Australian study (n=47) of older adults compared agreement between a self-reported UVR diary and dosimeters over 7 days, similar to our study (Spearman rank correlations r_s_=0.41; 95% CI 0.10, 0.64;  *P*=.01) [10]. The reliability and validity of sun exposure questions were compared to polysulphone dosimeter badges in 125 school children aged 14-15 years. Data were collected over 4 consecutive weekend days and the strongest Pearson correlation coefficient was between the questions “time in the sun”/”time spent outdoors” and dosimeters with r=0.52, *P*<.001 [21]. Glanz et al [22]  also found correlations between a self-reported UVR diary and 2 days of dosimeter measurements were fair to good in a US sample of lifeguards, parents, and children (n=515). In a US sample of radiologic technologists (n=124) the Pearson correlation coefficient between UVR diaries and dosimeters was high for northern (r=0.69,  *P*<.001) and southern (r=0.57,  *P*<.001) regions [23].

Our qualitative data showed paper-based diaries can be inconvenient and cumbersome to access and may not be completed in a timely manner (such as when backfilling diaries). Reported barriers for the paper diary in our study included requiring a pen, a surface to write on, flipping pages to view clothing coding, and inconvenience for travel. Overall, participants preferred the app over the paper diaries for recording UVR exposure for more than 1 week. However apps are not without problems of their own, with 42% (8/19) of participants in our study experiencing technical barriers when accessing the UVR app. Technical software support should be available for participants during the intervention period. Most of the barriers encountered were easily fixed by the participant (ie, insufficient phone battery; smartphone update required). There were advantages of the app for the research team. The data collected from participants in the UVR app was exported into the analysis software and reduced the staff workload required for data entry. There were 13 variables to input into the analysis software from the paper diary, which also required a 10% double data entry check quality control measure.

Our qualitative data showed recording personal UVR exposure on a daily basis made participants more sun aware and encouraged them to use more sun protection. Previous work by Koster et al [24] reported similar results, which showed using a UVR dosimeter or keeping a diary increased attention towards the behavior examined and therefore may influence this behavior. Consideration when designing studies with a measurement-only control arm should be taken in light of these findings. Interventions that use smartphones are increasingly used to improve adherence to preventive behavior [25,26], and the app diary could be embedded into these already electronic interventions.

We found participants under-reported the actual amount of low-intensity, but not moderate-to-vigorous–intensity physical activity compared to accelerometer data. This may be because accelerometers detect both incidental (ie, unstructured) and purposeful (ie, structured) physical activity. In contrast, participants may have only recalled their purposeful activity [27]. The low agreement observed was in line with studies published in the literature on physical activity, with a review of 148 studies reporting an average agreement of 0.37 (SD 0.25) [17]. The duration and intensity level of physical activity was captured in the online app. However, whether this activity was conducted “indoors” or “outdoors” should be included in future versions. This would allow for the tracking of time spent in sun-exposed, outdoor physical activity in future studies. Further extending the app to include a question on sunbed use would also be relevant to international settings.

### Limitiations

While this small study provided feasibility data, larger studies are required to further validate the app. Three dosimeters in the study malfunctioned due to technical error. Further limitations of this study were the self-reported outcome measures, which can be subject to recall and social desirability biases. The study used convenience sampling in a university setting recruiting a young age group, hampering generalization of the findings to the broader population. However, reducing excessive UVR exposure in young people is important for skin cancer prevention, as previous studies have shown that young people are at higher risk of sunburns, with adults aged 18 to 24 years 7 times more likely to report sunburn than those over 65 years. This group was therefore an appropriate initial target group for the use of the UVR app [28].

### Conclusion

Technology advances have the potential to increase the reach and impact of prevention programs. Our study demonstrates self-report using an app can result in reliable and convenient personal UVR exposure data collection. There are several advantages to recording UVR exposure in an app notably: 1) functions to alert and remind users to input data, thereby reducing missing data, 2) direct data entry by participants to eliminate data entry errors when paper diaries are transferred to an electronic database, which have been previously reported [29], 3) the ability to monitor daily data entry compliance, 4) minimizing the risk of participants losing the paper diary, and 5) streamlining the analysis process.

## Appendix

Multimedia Appendix 1 Ultraviolet Radiation (UVR) app.

Multimedia Appendix 2 Example of Paper Sun Diary adapted from previous studies [9,18]. A) Sun diary clothing and physical activity guide. B) Daily sun diary entry sheet.

## Abbreviations

SPF: sun protection factor
UVR: ultraviolet radiation
