# Supplementary material for: Capturing Ultraviolet Radiation Exposure and Physical Activity: Feasibility Study and Comparison Between Self-Reports, Mobile Apps, Dosimeters, and Accelerometers
Source: JMIR Res Protoc. 2018 Apr 17;7(4):e102. doi: 10.2196/resprot.9695 (PMC5930172; doi:10.2196/resprot.9695)
Supplement: Multimedia Appendix 2 [file resprot_v7i4e102_app2.pdf]

Multimedia Appendix 2 Example of Paper Sun Diary adapted from [9,18]. A) Sun diary clothing and physical activity guide. B) Daily sun diary entry sheet.

A)

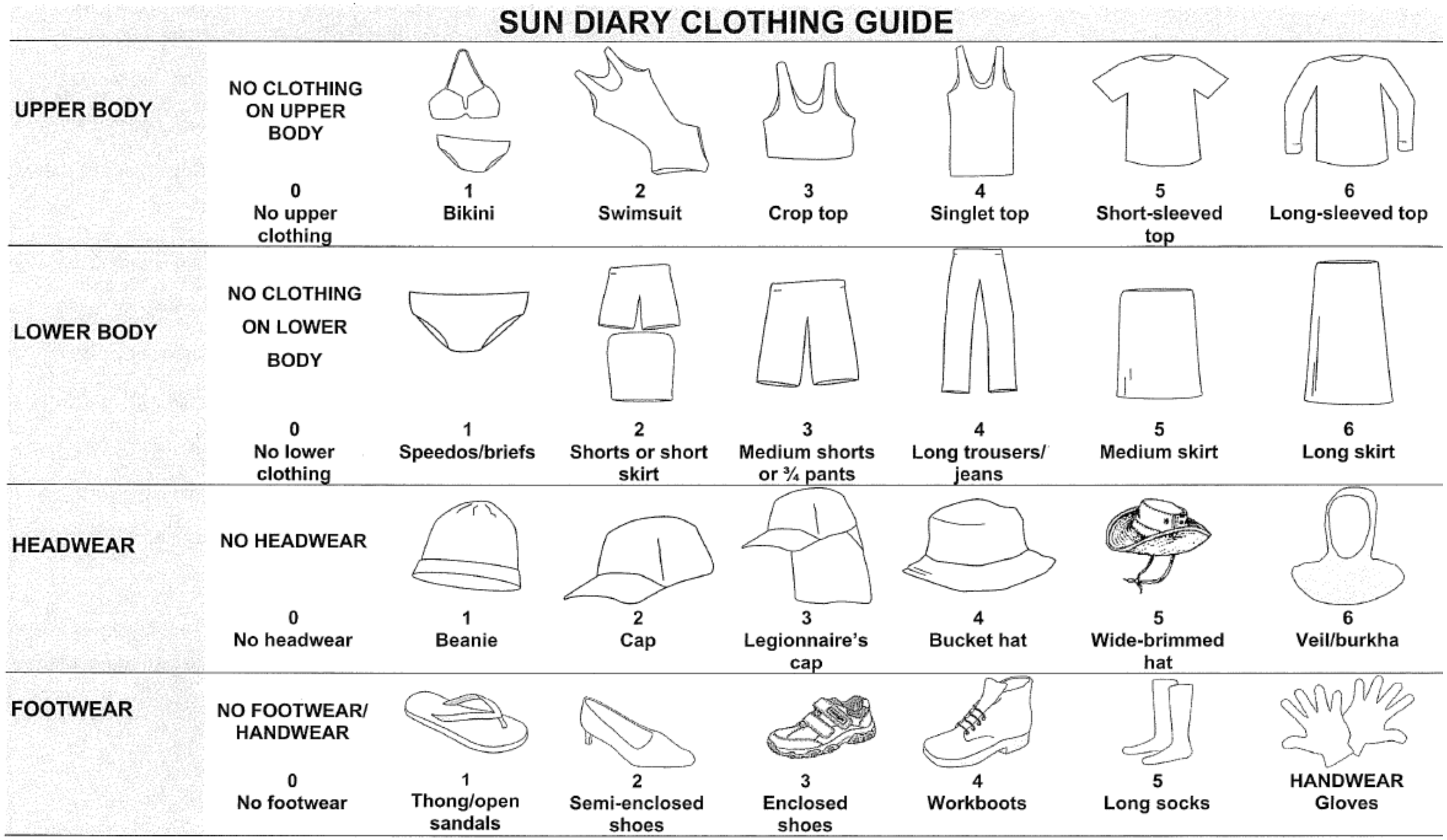

SUN DIARY CLOTHING GUIDE

**UPPER BODY**	**NO CLOTHING ON UPPER BODY** **0** No upper clothing	**1** Bikini	**2** Swimsuit	**3** Crop top	**4** Singlet top	**5** Short-sleeved top	**6** Long-sleeved top
**LOWER BODY**	**NO CLOTHING ON LOWER BODY** **0** No lower clothing	**1** Speedos/briefs	**2** Shorts or short skirt	**3** Medium shorts or ¾ pants	**4** Long trousers/ jeans	**5** Medium skirt	**6** Long skirt
**HEADWEAR**	**NO HEADWEAR** **0** No headwear	**1** Beanie	**2** Cap	**3** Legionnaire's cap	**4** Bucket hat	**5** Wide-brimmed hat	**6** Veil/burkha
**FOOTWEAR**	**NO FOOTWEAR/ HANDWEAR** **0** No footwear	**1** Thong/open sandals	**2** Semi-enclosed shoes	**3** Enclosed shoes	**4** Workboots	**5** Long socks	**HANDWEAR** Gloves

PHYSICAL ACTIVITY CODES
0 = No physical activity
1 = Mild physical activity (for example, walking to travel from place to place, and walking that you might do solely for recreation, sport, exercise, or leisure)
2 = Moderate physical activity (for example, carrying light loads, bicycling at a regular pace, slow jogging, or doubles tennis) ***Please do not include walking***.
3 = **Vigorous physical activity** (for example, heavy lifting, digging, aerobics, running, swimming, or fast bicycling)
***PLEASE DO NOT INCLUDE DRIVING AS A PHYSICAL ACTIVITY.**

B)

DAY 1

Date ☐☐ / ☐☐ / ☐☐☐☐ Participant ID: ☐☐☐☐

UV Badge Number: ☐☐☐☐☐ Interview Number: ☐

	TIME OUTDOORS IN THE SUN (Cross the box which best represents the amount of time that you spent in the sun during each one hour interval shown below)					Did you apply sun-screen?	Where did you apply sunscreen?	SPF of Sunscreen	TYPE OF CLOTHING WORN Please refer to Clothing Guide (You must write one number in each box)					LEVEL OF PHYSICAL ACTIVITY	
	0 mins	<15 mins	15-29 mins	30-44 mins	45-60 mins	Cross all that apply Leave blank if not used	1=ALL exposed body sites 2=MORE THAN HALF of exposed body sites 3=LESS THAN HALF of exposed body sites	(number only, omit '+')	Upper body	Lower body	Head wear	Foot-wear	Gloves (Cross all that apply) Leave blank if not used	0 = None 1 = Mild 2 = Moderate 3 = Vigorous (You must write one number in each box)	
**Morning**														Indoors	Outdoors
5 - 6 am															
6 - 7 am															
7 - 8 am															
8 - 9 am															
9 - 10 am															
10 - 11 am															
11 - 12 am															
**Afternoon**															
12 - 1 pm															
1 - 2 pm															
2 - 3 pm															
3 - 4 pm															
4 - 5 pm															
5 - 6 pm															
6 - 7 pm															

Did you make sure that your wristband was not covered by clothing today? ☐ **Yes** ☐ **No**

Please document any problems you had with the UV badge (e.g. The badge was covered by clothing for approx 30 minutes while I was outside at midday; the badge fell off at 3pm; the badge got wet etc)
